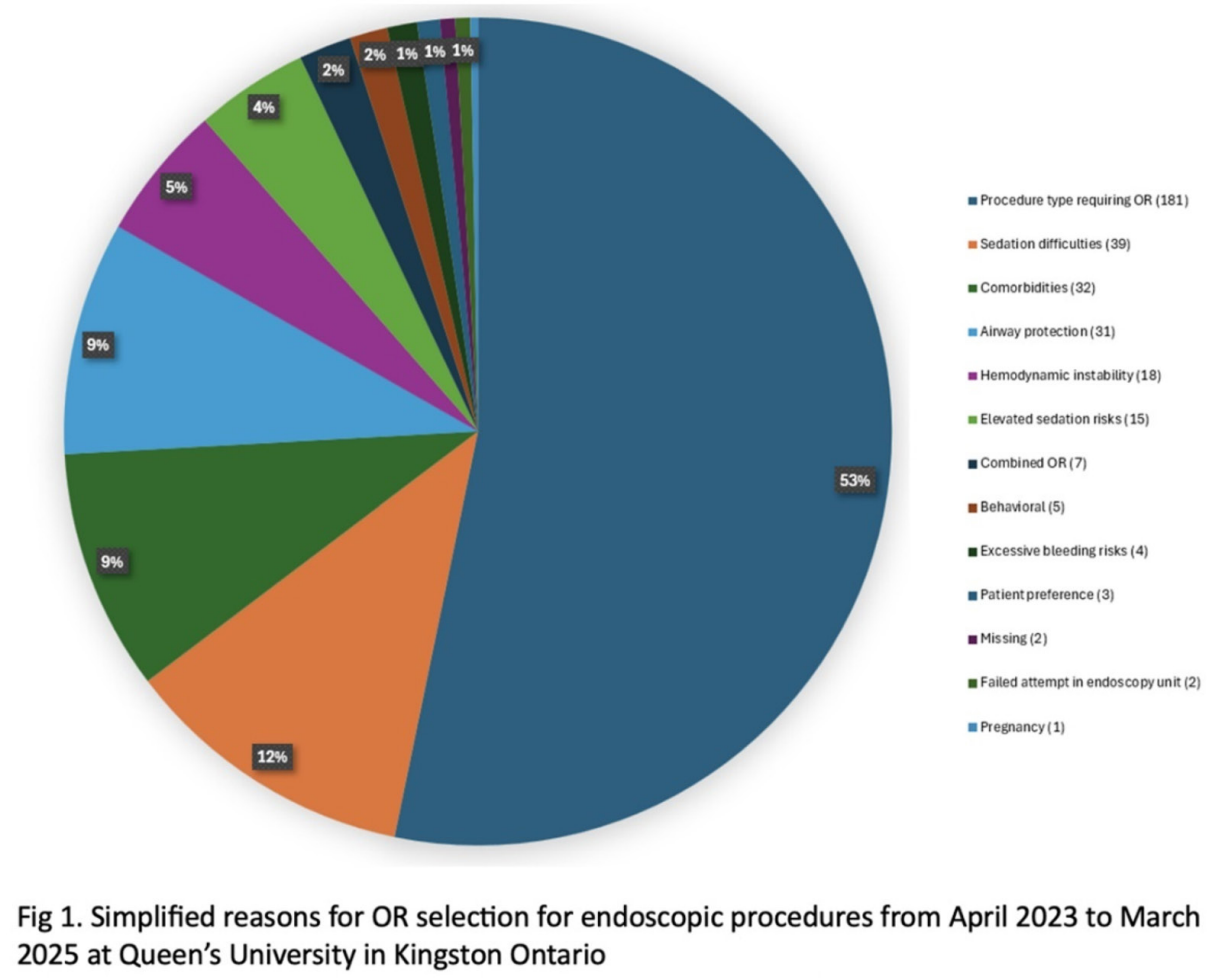# Poster Session I - A145 RETROSPECTIVE SINGLE CENTER REVIEW OF ENDOSCOPIC PROCEDURES REQUIRING ANESTHEISA ASSISTANCE IN THE OPERATING ROOM

**DOI:** 10.1093/jcag/gwaf042.145

**Published:** 2026-02-13

**Authors:** K Moss, R Arya, R Hindocha, R Bechara, M Rai

**Affiliations:** Medicine, Queen’s University, Kingston, ON, Canada; Medicine, Queen’s University, Kingston, ON, Canada; Medicine, Queen’s University, Kingston, ON, Canada; Medicine, Kingston Health Sciences Centre, Kingston, ON, Canada; Medicine, Kingston Health Sciences Centre, Kingston, ON, Canada

## Abstract

**Background:**

Sedation for GI endoscopy ranges from moderate (conscious) to deep sedation or general anaesthesia (GA). Most procedures, both inpatient and outpatient are completed in the endoscopy unit. However; some patients, or especially complex procedures, require GA with intubation, which at our institution necessitates the procedure to be completed in the operating room (OR). Additionally, as endoscopic techniques continue to advance and become more aligned with surgical techniques, anesthesia requirements will likely increase.

**Aims:**

This retrospective study aimed to identify patient and procedural factors associated with performing endoscopic procedures in the OR instead of the endoscopy unit at Kingston Health Sciences Centre (KHSC).

**Methods:**

Data from all adult GI endoscopic procedures performed from April 2023 to March 2025 at KHSC (Kingston General Hospital site) were collected, and only procedures conducted in the OR were analyzed. The procedure type, indication, urgency level, patient characteristics, and reason for OR was collected.

**Results:**

Of 3,607 GI endoscopic procedures, 340 (9%) were performed in the OR. Of these cases, 247 (73%) were advanced therapeutic procedures such as endoscopic retrograde cholangiopancreatography (ERCP), endoscopic submucosal dissection (ESD), stenting, and peroral endoscopic myotomy (POEM). The mean age of patients was 60, SD 18. There were 182 (54%) males. There were 35 (10%) procedures that had a failed conscious sedation attempt and 43 (13%) of patients were on chronic opioid therapy. The mean ASA was 3.3, SD 0.74.

Among inpatient cases (n = 144), the three most common procedures were upper endoscopy (49 cases, 34%), ERCP (27, 19%), and esophageal stent placement (11, 8%). Of the 196 (58%) outpatient procedures the top three were ESD (95, 48%), POEM (41, 21%), and ERCP (20, 10%). The most common reasons for OR for emergent cases was hemodynamic instability, (13, 18%), for elective inpatient it was comorbidities (5, 13%), and for outpatients, ESD (54, 28%). Of the 38 elective inpatients and the 190 outpatients, 9 (24%) and 25 (13%) were on home narcotics and 24 (63%) and 103 (54%) had significant comorbidities respectively based on physician consultation.

**Conclusions:**

There are many reasons for procedures to be completed with anesthesia support. At our centre, all anesthesia-supported endoscopic procedures are performed in the OR due to infrastructure constraints. Our review shows that advanced therapeutic procedures, failed conscious sedation, and significant comorbidities are the primary drivers of OR use. These findings highlight the importance of planning anesthesia resources and considering models that would allow select complex cases to be safely managed in the endoscopy unit. This study is limited by its design as a retrospective chart-review and future prospective studies are required.

**Funding Agencies:**

None